# Physical and behavioural health of dogs belonging to homeless people

**DOI:** 10.1017/awf.2024.12

**Published:** 2024-02-26

**Authors:** Camille King, Thomas J Smith, Kyle Kabrick, Amy Dzur, Temple Grandin

**Affiliations:** 1 Canine Education Center, LLC, Loveland, Colorado 80537, USA; 2 Northern Illinois University College of Education, Dekalb, Illinois, USA; 3 VA Eastern Colorado Health Care System, Aurora, Colorado, USA; 4 Colorado State University, Fort Collins, Colorado, USA

**Keywords:** animal welfare, companionship, dog health, One Welfare, homelessness, human-animal interactions

## Abstract

Homeless persons with dogs are often the subject of stigma, with the public criticising them for not having a proper lifestyle to care for a pet. There is solid documentation of how dogs enhance a homeless person’s life, but there are few publications that address the welfare of the dog. This descriptive study assesses the physical and behavioural health of dogs belonging to homeless persons through a One Welfare lens by observing animal/human well-being, environment, and “a life worth living”. A survey was carried out along with a visual assessment of the condition of the dog for 100 human-dog dyads in the Western United States. Results showed that dogs of homeless persons were well cared for and physically healthy (which was consistent with other studies), and had few behavioural problems, but did display evidence of separation distress while the owner was away. Results from this study can provide information that may lead to policy and practice changes, including, for example, changes to policies and practices prohibiting dogs from being kept with their owner while staying at a homeless shelter. Typically, shelters report that they do not have the resources to care for a person with a dog.

## Introduction

Approximately 582,462 people were experiencing homelessness in the United States in 2022. This amounts to 18 out of every 10,000 people (National Alliance to End Homelessness 2023). As in individuals/families with homes, dog ownership may play an important role in the lives of these homeless persons.

Estimates of the percentage of homeless persons in the United States who have a pet dog have varied across studies. The Henwood *et al.* (2020) survey, carried out in 2019, reported 12% of homeless persons had a dog, while the National Alliance to End Homelessness (2020) survey reported that this percentage could range from 10–25%. Homeless persons (US) are often stigmatised by the public, who criticise them for not harbouring a ‘proper’ lifestyle — e.g. a home or a job — to care for a pet (Irvine *et al*. 2012; Kuhlenbeck 2021). Although Kerman *et al.* (2020) found that educational interventions will help to reduce public stigma regarding the relationship between homelessness and pet ownership in the US/Canada, dog ownership among homeless persons may confer benefits to the owner regardless of public perception. Irvine (2013a,b), for example, found that homeless persons view their dogs as beneficial in helping them to overcome adversity. There is much additional research on the benefits conferred to homeless persons from having a pet dog, such as a reduction in loneliness, improved mental health, and reduction in risk behaviours, such as substance use (Cleary *et al.* 2021; Scanlon *et al.* 2021a) but compared to literature focusing on the homeless individuals themselves, the literature addressing the welfare of the dogs owned by homeless people is scant.

The human-animal bond is a strong component within the homeless person-dog dyad. Homeless persons describe pets as ‘kin’, feel responsible for their pet, and report anticipatory grief when thinking about a future without their pet (Scanlon *et al.* 2021a). Cleary *et al.* (2021) observed that the benefits of pet ownership among persons experiencing homelessness included decreased social isolation and improved mental health. Other research (Rhoades 2015) has found that most homeless pet owners believe that their pets kept them company and allowed them to feel loved. In fact, many people in Rhoades’ study turned down housing opportunities because pets were not allowed in the facility they desired. This latter fact also highlights the fact that, although dogs confer considerable benefits to homeless persons, they also present lifestyle barriers. Rhoades reported that 49.2% of their sample said pets made it harder to stay in a shelter and 15.8% said it was more difficult to secure housing.

The aforementioned studies underscore the benefits and challenges of pet ownership among homeless people. But there are few corresponding studies that address the physical and behavioural health of dogs owned by homeless persons. Canine physical health can be assessed through the lens of the ‘Five Freedoms’ of animal welfare: thirst/hunger, physical/thermal discomfort, pain, fear/distress, and the ability to express normal behaviour (Coria-Avila *et al.* 2022) and, more recently, through the lens of the ‘Five Domains’ of animal welfare: nutrition, environment, health, behaviour, and emotional state, along with a life worth living (Webster 2016). Good canine behavioural health is characterised by a well-socialised dog with plenty of exposure to people, animals, new places, and fun experiences that allow for adequate mental stimulation and enrichment (Winkle *et al.* 2020; American Society for the Prevention of Cruelty to Animals [ASPCA] 2023).

Differences in dog characteristics between dogs owned by homeless vs non-homeless persons were examined by Williams and Hogg (2016), who conducted a study in the UK comparing 50 dogs owned by homeless persons to 50 dogs owned by persons living in a home (i.e. non-homeless persons). A questionnaire was completed, and a clinical health check was performed on the dogs. Visual assessment, palpation of the ribs and abdomen, and cardiac auscultation were carried out with each dog. It was found that dogs owned by homeless persons were significantly younger than those owned by non-homeless individuals. Most homeless dog owners fed their dogs a dry food diet and walked with their dogs several hours per day, while non-homeless pet owners tended to provide a mix of dry and wet food and, on average, walked their dogs twice a day for 20 min, but this varied depending on work schedules and social engagements. Homeless persons’ dogs were described as friendly, quiet, and without notable behavioural problems, such as aggression toward strangers or separation anxiety, while non-homeless persons’ dogs were more likely to be hyperactive or disobedient – (i.e. not following through with owner requests), display barking or destructive behaviour, and have house-soiling issues. Dogs owned by those with homes had a higher incidence of obesity than homeless individuals’ dogs (Williams & Hogg 2016).

In another study based in the UK with a sample of 21 dogs, it was found that homeless persons’ dogs were not as disadvantaged as the public believed (Scanlon *et al.* 2021a). The exercise level of dogs owned by homeless and non-homeless persons was equivalent while, consistent with Williams and Hogg’s (2016) findings, obesity was present in homeless persons’ dogs, but occurred at a lower prevalence than in non-homeless persons’ dogs. Scanlon *et al.* (2021b) study results did show 69% separation-related stress in dogs belonging to homeless persons.

Research assessing the population of dogs owned by homeless persons can easily be aligned with the ‘One Welfare’ approach. This approach encourages interdisciplinary collaboration and promotion of three areas: the well-being of animals; people; and their social environment (Pinillos 2018; One Welfare 2023). This animal welfare philosophy endorses minimising negative experiences, which can be challenging when shelter/housing is unstable and fostering a “life worth living” (Mellor 2016; Webster 2016). Animal welfare impacts human well-being through the human-animal bond and companionship. As noted in prior research, animal companionship reduces loneliness in humans and improves mental health. People worry less when their pet is in good health. Homeless persons have opportunities to maintain good health for their dog by collaborating with the free veterinary services clinics across the US (Street Dog Coalition 2023).

The current study used survey and observation techniques to gather information about dogs owned by homeless persons in the Western United States, with the goal of providing preliminary descriptive information about the physical and behavioural health of the dogs and the perceptions of their owners towards them. Although studies on dogs belonging to homeless persons have been carried out in the UK (Scanlon *et al.* 2021b), there are no known studies pertaining to the welfare of dogs belonging to this population in the US. Additionally, although sample size is not large due to the difficulty of accessing this population, it employs a larger sample size than prior studies.

This study sought to address the following research questions:

- What were the health and behaviour characteristics of dogs belonging to homeless persons?
- What were homeless persons’ perspectives on dog ownership?

## Materials and methods

Research study approval for the current study was obtained through the Office of Research Compliance, Integrity & Safety at Northern Illinois University (Protocol #HS22-0273). Informed consent to participate in the study was provided by each participant. Each participant provided responses to a survey addressing multiple factors of dog ownership (e.g. experience with dogs, where the dog was obtained, opportunities for exercise, etc). Each dog was present with the owner during data collection, and data were collected by a professional who also visually observed the physical health and behaviour of the dog, breed type, attachment to owner, among other characteristics. The survey items and observation prompts are included in the Supplementary material.

### Sample

The sample consisted of 100 human-dog dyads located in the Western United States. The study was conducted from May 2022 through to December 2022. Recruitment was accomplished by directly approaching participants, either on the street, in homeless encampments, at homeless military veteran dog events in the community, or through scheduled veterinary appointment times at the Street Dog Coalition at the Murphy Center in Fort Collins, Colorado. The semi-established homeless encampments included small tents or tarp shelter configurations. There were designated side streets in which homeless people parked their vehicles. Most parked vehicles were near a homeless resource centre and/or food bank. In each of these locations, we approached every visible human-dog dyad, administered the survey, and observed the dog and its interactions with the owner. Each person who agreed to participate completed the entire survey.

### Instrumentation

A 21-item research-constructed questionnaire was completed along with a visual assessment of the dog’s overall appearance, physical condition, and behavioural presentation made by the researchers. Visual physical assessment was conducted by a researcher trained as a veterinary nurse, and behavioural assessment was conducted by a certified applied animal behaviourist. One of the co-authors (TG) is an animal welfare expert. The physical health of the animal was based loosely on Body Condition Scoring (BCS) (Laflamme 1997) and visual notation of whether the animal was ‘underweight’ (rib cage, spine, or hips visible, lack of fat padding between skin and bones, ‘overweight’ (heavy fat padding around ribs, swinging waist, waddling gait, poor tolerance of exercise), or ‘normal weight’. The dog was also observed for any signs of physical illness such as eye or nose drainage, difficulty breathing, hair coat abnormalities such as hair loss, mange, or evidence of parasites (e.g. fleas or ticks). Lameness or gait abnormality was assessed when the dog was observed walking. Low energy or listlessness was evaluated during the visual assessment as a potential indicator of problems, such as fever, bradycardia, etc. Also, each dog was visually assessed for attachment to the owner (e.g. dog following its owner, licking owner, staying close to owner, watching owner, positive interaction with owner, showing distress when owner was not near), presentation of collar and tags, breed, and size (based on breed-standard height).

### Analysis

Descriptive statistics, including frequency distributions, percentage point estimates, and “exact” (Clopper & Pearson 1934) 95% confidence intervals for these estimates were computed for the sample based on responses to the closed-ended survey items. Chi-squared tests were used to assess relationships between several categorical responses, using a decision criterion of α = 0.05.

For open-ended survey items, the 1–2-word responses were evaluated by CK and categorised using expert judgment based on 32 years of professional experience working with dogs. For example, if a dog pulled on leash or jumped up on others, this behaviour was categorised as obedience/training; dog behaviour that included baring teeth, growling, or lunging at a person was categorised as aggression. A co-author, TG, (who is an animal behaviour specialist/animal welfare expert) then independently assigned each response to one of the categories identified by CK. The few discrepancies that occurred between the CK and TG were noted, discussed, and resolved.

## Results

### Owner responses to closed-ended survey items

Table 1 shows the distribution of owner responses to closed-ended items on the questionnaire, reflecting characteristics of the sampled dogs and their owners. Among the sampled dogs, slightly more than half (52%) were male, and an equal percentage (52%) were neither spayed nor fixed. A dog’s status as intact was not related to the dog’s sex [ 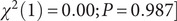 , nor was it related to whether the dog displayed tags [ 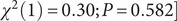 . Nearly half of the dogs (49%) were under four years of age. Most of the dogs (64%) were described by their owners as “active”. Many dogs (78%), however, were reported by their owners to show symptoms of separation distress. Overall, owners reported their dogs as being friendly, with 79% of dogs reported to show positive reactions to strangers, while only 2% were reported to show negative reactions.

**Table 1.** Relative frequency distribution of owner responses to closed-ended survey items (n = 100)

Characteristic		%	95% CI
Dog’s sex	Female	48%	(38%, 58%)
	Male	52%	(42%, 62%)
Dog is spayed or neutered	Yes	48%	(38%, 58%)
	No	52%	(42%, 62%)
Dog’s age in years	Less than 1 year	20%	(13%, 29%)
	1 to 3.99 years	29%	(20%, 38%)
	4 to 9.99 years	42%	(32%, 52%)
	10 years or older	9%	(4%, 16%)
Owner’s perception of dog’s role	Protection	3%	(1%, 9%)
	Companionship	59%	(49%, 69%)
	Both protection and companionship	38%	(28%, 48%)
Owner’s perception of dog’s activity level	Active	64%	(54%, 73%)
	Non-active	36%	(27%, 46%)
Owner previously owned an abused dog	Yes	75%	(65%, 83%)
	No	25%	(17%, 35%)
Owner had a dog as a child	Yes	94%	(87%, 98%)
	No	6%	(2%, 13%)
Owner brings dog to dog parks	Yes	64%	(54%, 73%)
	No	36%	(27%, 46%)
Dog exhibits separation distress	Yes	78%	(69%, 86%)
	No	22%	(14%, 31%)
Dog’s reaction to unfamiliar people	Mostly positive	81%	(72%, 88%)
	Mostly negative	3%	(1%, 9%)
	Mostly neutral	16%	(9%, 25%)

Among the dog owners, nearly all (92%) reported having had a dog as child, and 75% reported having at some point in their lives owned a dog that had been abused. A majority of owners (64%) reported taking their dog to a dog park. Very few dog owners (3%) said they owned their dog solely for protective purposes. Most (59%) reported owning their dog for companionship, while 38% said they owned their dog for both protection and companionship. The purpose of dog ownership (protection vs companionship) was not related to the dog’s observed size [ 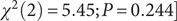 , nor to its status as a ‘bully breed’ [ 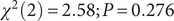 ].

### Responses to open-ended survey items

Table 2 presents a summary of responses to the open-ended survey items, where the responses were coded by two raters. As these results show, owners reported obtaining their dogs primarily from family/friends (47%), animal shelters or rescue organisations (25%). Other sources (e.g. breeders, advertisements, such as Craigslist) were less common, and 15% of owners reported finding their dog as a stray. Bully (24%) and Toy breeds (23%) were most common, although Sporting (15%), Working (14%), Herding (11%), and Terrier (8%) breeds were also well-represented. The Non-Sporting breeds constituted 2% of the sample. Most participants reported that they either lived with their dog in a tent encampment (47%) or in their vehicle (45%). Several participants (8%) reported that they “couch-surfed” at a friend’s apartment or stayed at a homeless shelter, but also noted that this was difficult because it required them to keep their dog elsewhere. If the owner was unable to care for their dog, most reported that either a relative/partner (53%) or a friend (41%) could do so. Interestingly, 6% of participants said they never left their dog with others and had no contingency plan should they become hospitalised and/or unable to care for their dog. When pressed for a response, they responded that they perhaps could temporarily leave the dog in a humane society. Only 5% of owners reported experiencing a serious, life-threatening medical emergency with their dog, and even minor medical emergencies (15%) were relatively infrequent. The vast majority (80%) reported no medical emergencies.

**Table 2.** Relative frequency distribution of owner responses to open-ended survey items (n = 100)

Characteristic		Percentage	95% CI for percentage
Where dog was obtained	Friend/family	47%	(37%, 57%)
	Shelter or rescue organisation	25%	(17%, 35%)
	Found	15%	(9%, 24%)
	Breeder	3%	(1%, 9%)
	Advertisement	10%	(5%, 18%)
Current housing situation	Tent encampment	47%	(37%, 57%)
	Vehicle	45%	(35%, 55%)
	‘Couch-surfing’ or homeless shelter	8%	(4%, 15%)
Reason for owning dog	Rescued from difficult situation	21%	(13%, 30%)
	Wanted companion	34%	(25%, 44%)
	Loves dogs	15%	(9%, 24%)
	Emotional support	13%	(17%, 21%)
	Breeding	11%	(6%, 19%)
	Gifted to owner	6%	(2%, 13%)
Behaviours owner wishes to teach or change	Obedience cues	31%	(22%, 41%)
	Separation distress/fear issue	10%	(5%, 18%)
	Dog-to-dog aggression	7%	(3%, 14%)
	Defence/resource aggression	5%	(2%, 11%)
	Predatory aggression	5%	(2%, 11%)
	Barking behaviour	4%	(1%, 10%)
	None	38%	(28%, 48%)
Who cares for dog when owner is unable	Friend	53%	(43%, 63%)
	Relative/partner	41%	(31%, 51%)
	Never leave dog with others	6%	(2%, 13%)
What dog eats	Multiple brands of dry dog food	68%	(58%, 77%)
	Whatever food is available	23%	(15%, 32%)
	Combination of wet and dry dog food	9%	(4%, 16%)
Obstacles to dog ownership	Shelter	28%	(18%, 35%)
	Transportation	4%	(1%, 10%)
	Both shelter and transportation	3%	(1%, 10%)
	Other	16%	(9%, 25%)
	None	51%	(41%, 61%)
Dog Emergencies Life Threatening	Problem	5%	(2%, 11%)
	Minor problem	15%	(9%, 24%)
	No emergencies	80%	(71%, 87%)
Dog-to-dog interactions	Positive	78%	(69%, 86%)
	Negative	17%	(10%, 26%)
	Neutral	5%	(2%, 11%)
Breed	Bully	24%	(16%, 34%)
	Toy	23%	(15%, 32%)
	Sporting	15%	(9%, 24%)
	Working	14%	(8%, 22%)
	Herding	11%	(6%, 19%)
	Terrier	8%	(4%, 15%)
	Hound	3%	(1%, 9%)
	Non-sporting	2%	(0%, 7%)
One word that describes your dog	Character	28%	(19%, 38%)
	Companionship	16%	(9%, 25%)
	Familial relations	7%	(3%, 14%)
	Behavioural presentation	20%	(13%, 29%)
	Emotion	27%	(19%, 37%)
	Miscellaneous other	2%	(0%, 7%)

When obstacles to ownership were considered, securing housing (26%), and obtaining access to public transportation (4%), or both (3%) were the main problems that homeless people reported in owning a dog. Typically, homeless shelters do not allow dogs within their facilities. Participants also reported that they were unable to bring their dog onto a bus or train unless they were able to provide proof that the dog was a service dog. (Note: In the US, emotional support dogs do not meet the criteria for status as a service dog). Some participants reported other obstacles to having a dog, such as a dog with anxiety/fear, obedience issues, and medical problems such as tick infestations or poor dental health.

The most common behaviours that owners wanted to instill or change in their dogs were learning obedience cues (31%), and reducing behaviours related to anxiety/fear (10%). Although participants reported that their dog showed separation distress while they were away, they claimed it not to be an overwhelming concern since they limited their time away from the dog, i.e. if they went into a shelter quickly to get a meal, or they would have another person care for their dog for that short time. Owners also reported wanting to decrease various types of aggressive behaviour, including dog-to-dog aggression (7%), defence/resource aggression (5%), and predatory aggression (5%). Negative dog-to-dog interactions, however, were reported by owners as relatively uncommon (17%), and most owners described their dog’s interaction with other dogs as either positive (78%) or neutral (5%).

When asked for the reason why they took ownership of their dog, the most common response (34%) was for companionship, and another 21% took ownership to rescue their dog from a difficult situation. Others reported owning their dog for emotional support (13%), or because they “love dogs” (15%), while others (11%) wanted their dog for breeding purposes. Some owners (6%) took ownership because the dog was given as a gift.

Participants were asked to provide a single word that best described their dog. There was great variation in responses, and we organised the words into the following themes: (1) words related to *character* (28%) such as “charismatic” or “follower”; (2) words related to *companionship* (16%) such as “friend” or “buddy”; (3) words related to *familial relationships* (7%) such as “my baby” or “my kid”; (4) words related to *behavioural presentation* (20%) such as “tenacious,” “playful,” “hyperactive,” or “fearful”; and (5) *emotion* words (27%), such as “love,” “loving,” or “happiness”.

### Results pertaining to researchers’ visual observation of dogs

Table 3 shows results from visual observations of dogs by the researchers. As shown, a majority of (51%) were estimated by the researchers to be large-sized, while 29% were small-sized and 20% medium. Mostly (77%) dogs were evaluated as being a healthy weight, while 18% were overweight and 5% underweight. Similarly, a large percentage of dogs (93%) were evaluated as physically healthy and not showing symptoms of illness. Visual observation of each dog’s behaviour showed most to be friendly (68%) or neutral (21%) in disposition, while relatively few were reported to be either shy (6%) or aggressive (4%). This was largely in accordance with owners’ descriptions of their dogs, as 79% reported their dog as showing positive reactions to strangers, while only 2% of dogs were noted to show negative reactions. All dogs (100%) were observed by the researchers as showing attachment to their owners.

**Table 3.** Relative frequency distribution of dog characteristics based on visual observation (n = 100)

Characteristic		Percentage	95% CI for percentage
Dog observed as attached to owner	Yes	100%	(96%, 100%)
	No	0%	(0%, 4%)
Dog’s observed disposition	Friendly (tail wagging)	68%	(58%, 77%)
	Shy (cowering)	6%	(2%, 13%)
	Threatening (growling or aggressive baring of teeth)	4%	(1%, 10%)
	Neutral	21%	(13%, 30%)
	Other (barking)	1%	(0%, 5%)
Dog’s observed body appearance	Healthy weight	74%	(68%, 85%)
	Overweight	14%	(11%, 27%)
	Underweight	5%	(3%, 14%)
Dog displays sickness symptoms*	Yes	7%	(2%, 12%)
	No	93%	(86%, 97%)
Dog displays collar	Yes	92%	(85%, 96%)
	No	8%	(4%, 15%)
Dog displays tags	Yes	43%	(33%, 53%)
	No	57%	(47%, 67%)
Dog size	Small	29%	(20%, 39%)
	Medium	20%	(13%, 29%)
	Large	51%	(41%, 61%)

* Sickness symptoms – cherry eye, hair loss, eye infection, foreign body in paw.

## Discussion

Two prior studies (Williams & Hogg 2016; Scanlon 2021b) — both conducted in the UK — focused on homeless persons with dogs, and findings from the current study were in accordance with this research. In particular, the physical and behavioural well-being of dogs belonging to homeless persons was, overall, positive. This occurred despite concerns to the contrary that have been reported from studies of non-homeless persons (Irvine *et al.* 2012; Kuhlenbeck 2021).

Contrary to the findings of Williams and Hogg (2016), our study showed separation distress to be a common problem, with over three-quarters of owners reporting it as an issue. Our study showed evidence of separation distress when the owner was away from the dog. This differs from Williams and Hogg who reported no separation distress. Interestingly, however, results from our study were consistent with Scanlon *et al.* (2021b), who found evidence of separation-related distress in 69.1% of the sample. The Scanlon *et al.* study, however, had a much smaller sample of dogs (n = 21) compared to ours (n = 100). It is important to note that our study was conducted in the US while both Williams and Hogg and Scanlon *et al.* were studies conducted in the UK. Location and cultural norms may provide one reason for the variation in study findings.

Respondents reported that their dogs presented increased vocalisation and destruction in vehicles or in tents, when the owner was not present, especially when the dog was unable to establish the line-of-sight visual status of the owner. These separation distress behaviours would appear plausible since the dog typically remains in continuous contact with the homeless person and any break to that routine could cause the dog distress. There are times when a homeless person will go into a facility to obtain a meal while the dog is left in a vehicle or tent. This behaviour can impact the animal welfare issue of fear/distress. This, of course, is not unique to the homeless population as dogs living in homes with non-homeless people can experience similar levels of distress when the owner has departed for an appointment or some other activity. It is important to educate dog owners regarding the stress an animal can experience when alone and how they might work with their animal to help alleviate such problems.

In keeping with the findings of Williams and Hogg (2016) and Scanlon *et al.* (2021b), the physical condition of homeless persons’ dogs was very good. A relatively small percentage were overweight or obese with an even smaller percentage underweight (and one of the underweight dogs was nursing a litter of puppies). A dog’s weight status (overweight, underweight, healthy weight) was independent of the breed size (small, medium, or large), 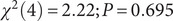 . Study participants reported that their dogs were active, which can help to maintain a healthy weight. Anecdotally, many participants reported having to walk many miles each day with their dog to travel to different facilities for a meal, to the food bank, to a homeless shelter, or another facility with homeless-dedicated resources. Two older toy breeds presented with a degree of hair thinning/loss. These two dogs were being evaluated through Street Dog Coalition – a programme that provides free veterinary care, and for which many owners in the present study expressed gratitude. None of the dogs in the sample appeared dirty. Most had hair coats that gave the appearance of being groomed, which was surprising given that they were frequently out in the community and exposed to the elements. Consistent with prior research, the dogs were mostly fed high nutritional quality dry dog food obtained from pet food banks. A smaller proportion of the survey participants reported feeding their dog whatever food they ate themselves, but they assured the researcher that their dog’s needs were met before their own. Veterinary emergencies, when they occurred, were triaged as either a critical or life-threatening event, such as a cardiac or respiratory insult, or a non-critical event, like a skin laceration or a fractured bone.

Many of the dogs in the sample were mixed breeds. Commonly, participants said they believed their dog was a particular breed (e.g. Chihuahua) or they reported being told by a rescue group or friend that the dog was a particular breed, despite the dog neither looking like the breed in question, phenotypically nor presenting behavioural traits typical for that breed. The researchers considered each owner’s idea of their dog’s breed, but for the purposes of this study, the dog was classified via evaluation of phenotypical presentation by a dog professional. Many participants stated that the American Pit Bull Terrier earns a bad reputation, but that they had found the Pit Bull to be quite friendly and a solid companion. They also reported that, as homeless individuals, they personally identified with the discrimination people exhibit towards the Pit Bull breed. Of the 100 dogs observed, Pit Bulls were the most commonly kept breed, owned by nearly a quarter of participants. A majority of these Pit Bulls (71%) were obtained from family/friends, while smaller percentages were obtained from rescue groups or shelters (17%), advertisements (8%), or simply were found as strays (4%).

Nearly all participants (92%) reported having a dog as a child, which might suggest that early childhood experiences serve as motivation for subsequent dog adoption in adulthood. Having a dog as a child may also be critical to the learned aspect of a positive perception of dogs as companions. Caring for a pet companion — particularly while in a homeless situation — could reduce loneliness, as identified in prior research (Cleary *et al.* 2021; Scanlon *et al.* 2021a). It is also notable that a non-negligible proportion of respondents (21%) indicated altruistic motives for dog adoption so, at least for some respondents, having a dog may fulfill needs beyond personal companionship.

One particular survey item pertained to whether a participant had previously owned a dog that had been abused. Although this was not part of any formal research question for this study, we had an interest in the participant responses because of a prior research study on abuse characteristics and shelter dogs (King *et al.* 2021). In the present study, approximately 75% of participants reported they had previously owned an abused dog. Interestingly, participants discussed that they believed their prior pet dog was abused because the dog would cower or, at times, urinate whenever a person attempted to pet the dog. Abuse is defined as violence or neglect perpetrated against dogs (ASPCA 2023). Kaldahl (2023) reported it to be difficult to determine whether a pet has been abused or neglected, as opposed to simply being under-socialised or genetically predisposed to exhibit fearful behaviour. Further, recent research (King *et al.* 2021) has found that, in the US, prospective dog owners are not dissuaded from adopting a dog that has been abused and may actually find some appeal in the prospect of such adoptions. Additional study would be helpful to explore this topic and address why people choose to adopt an abused dog and what education is needed to address socialisation and exposure to novelty when a dog does present body language consistent with fear.

We anticipated that homeless persons’ dogs would be well socialised because they are frequently interacting with people and other dogs in the community. Most dogs presented as friendly, socially approached the researcher, and often licked the researcher’s hand, and these behaviours were consistent with owner reports, where only a very small percentage of dogs showed negative reactions to strangers. In this study, a few dogs either barked or growled upon the researcher’s approach. The single dog in this study that growled during observation was lying next to its owner chewing a bone, thus the behaviour appeared to reflect resource guarding of a food item rather than being reactive to the stranger. Once the owner removed the bone, the dog sat down and was non-reactive toward the researcher. Two other dogs barked at the end of the leash upon the researcher’s approach, but then quickly calmed down when the owner directed the dog to do so. Of the dogs that were reported by their owner as showing neutral response to strangers (16%), all completely ignored the researcher’s approach or attempts to pet it. These ‘neutral’ dogs occasionally looked at the researcher while being petted, or lay down and appeared disinterested. One of these dogs fell asleep during survey data collection.

Nearly three-quarters of survey participants reported that their dogs had positive reactions to other dogs, and that this was evident in any type of dog-to-dog interaction or play situation. A moderately small proportion were reported to display negative reactions to other dogs. These included reports of older dogs not wanting to interact with other dogs; specifically, if the older dog was experiencing pain and an energetic dog approached, or dogs who had been in a dog fight in the past who would present avoidant or negative behaviours toward other unfamiliar dogs.

Although most dogs in this study appeared well-behaved, sitting quietly while visiting or walking next to the owner while on leash, nearly one-third of owners reported that their most desired behavioural change was for their dog to show better compliance with obedience cues. However, a slightly larger proportion believed their dog needed no behavioural training at all. Approximately one-third of owners wanted their dog to show less dog-to-dog aggression, resource-aggression, or predatory aggression. Several of these dogs were intact males that owners reported as showing dog-to-dog reactivity or a predatory response, such as, per report, chasing a squirrel up a tree.

Nearly half of owners reported obtaining the pet from a friend or a family member. Those who adopted a dog from a humane society reported providing a homeless shelter address or a family member’s address during the application process, because this was a requirement for adoption. Craigslist offered some animals for free adoption and sometimes the participant was given the dog by another homeless person who could no longer care for the animal. A handful of dogs were found as strays in the community or followed the homeless person while on a walk. Numerous stories were shared by participants about helping to care for an animal in a time of need, and the fact that this occurred when the participant struggled themselves with a disadvantaged lifestyle was commendable. For example, owners reported stepping forward and taking ownership and caring for a dog because the original owner had died, or adopting a dog that was going ‘kennel crazy’, or giving shelter in their vehicle to a dog that was freezing while sleeping on the ground with another homeless person.

In this study, visual observation was completed assessing each dog’s attachment to its owner. Attachment theory describes two behaviours as reflective of attachment: (1) maintenance of proximity between the two living entities; and (2) special behaviour between two animate entities that they would not exhibit toward other animate entities (Bowlby 1969; Nagasawa *et al.* 2009). Dogs were consistently observed as desiring closeness to their owners. Behaviourally, dogs provided eye contact with their owners, were tactile with their owners, initiated play bows with their owners, but these behaviours were not necessarily shared with other persons in the area.

State laws in the United States require pet owners to have their dog licensed. Approximately 92% of the dogs in our sample wore a collar. Licensing a pet dog provides proof of rabies vaccination, contact information for the owner should the dog become lost, and also provides some funding to local humane societies. When visually assessed, only 43% of dogs had visible tags on their collars. An implication for not displaying a tag is that the dog could be at risk of impoundment by authorities should a tag not be available if questioned. Clearly, it is important that education be provided to this population about the risks involved if tags are not displayed, particularly when the dogs are highly visible and regularly in outdoor settings, as these dogs typically were. In this study, when tags were not displayed, we discussed the potential implications with the owner. Additionally, contact information for the Street Dog Coalition was provided to all participants so that they could obtain free veterinary services and vaccinations for their pet.

The vast majority of the homeless persons in this study lived in a tent or a vehicle. Some of these living sites were not very clean, with refuse or old clothing strewn outside the tent or in the back seat of a vehicle. The dogs, however, seemed to be healthy and happy in their surroundings with adequate food and water, and appeared to receive much affection from their owners.

### Limitations and future research

This study relied upon overall visual appearance of health and owner reports to assess dogs’ physical health, behavioural health, and welfare. Without conducting laboratory tests, radiology, and neurological work-ups, it is difficult to conclusively verify the health status of a dog. Visual observation in field work is a limitation in itself. This type of observation is subjective since it is based on the individual’s personal interpretations of what is seen, heard, or felt. It was important in our study to make sure that the visual assessments were conducted by individuals experienced in veterinary as well as behavioural health. Another limitation is that homeless persons living in the roughest or most extreme conditions (e.g. sleeping under a shrub or living in very dangerous areas) were not included in our sample.

The current study was limited in sample size (n = 100), was a convenience sample, and also was limited to homeless persons living in tents or vehicles and residing in the Western United States. Thus, obtaining precise, nationally representative estimates of the proportions of various characteristics of the dogs and their owners is not realistic, and the nature of the sample (convenience sample) certainly has the risk of selection bias. Gathering data from even a sample of this size was challenging, and involved a great deal of travel, resourcefulness, and even luck. However, additional data from future studies might help to refine the estimates obtained in the current study, as well as provide additional depth and characterisation of this seldom-studied population. This study did not include data from a comparison sample on non-homeless persons, so direct inferences about how dogs belonging to homeless persons may differ from those owned by non-homeless persons could not be made. Additionally, as with any self-reported survey study, the validity of the obtained data was dependent on the recollections and truthfulness of respondents, although we had no reason to believe that the homeless population would differ from other populations in this regard. In fact, we were struck by the earnestness and sincerity of the study participants, and their passion, affection, and devotion towards their canine companions.

It is the researchers’ hope that the results from this study and future studies might provide insight into the sociability and health of these dogs, which could serve to soften policies, ordinances, and laws that operate as barriers to dog ownership among this population, and thus allow more homeless shelters to cater for individuals with dogs. Recently, grant support has been emerging for programmes that provide housing for homeless persons and their dogs (e.g. Homeward Hounds in Grand Junction, Colorado, US). These programmes and contexts, in addition to providing a valuable and much-needed resource for these individuals and their dogs, also might provide fertile ground for additional research on homeless persons and their dogs.

### Animal welfare implications

Our study population comprised dogs belonging to homeless persons. Overall, the welfare of these dogs was very good considering challenging living conditions. Of a sample of 100 dogs, 93 were in good physical condition. Of the seven dogs observed to have a type of health issue, five were being seen at Street Dog Coalition, a free veterinary service, for eye problems (3), ear infection (1), and foreign body (cactus spine) in a paw (1). This showed that homeless persons cared for their dogs’ welfare. Only one senior dog (14 years) had some hair loss and was living in an encampment. The owner pushed the dog in a stroller. One other dog had a tick observed on its scruff. No other external parasites were noted.

The findings from this study showed that a sizeable percentage of dogs (78%) experienced separation distress. This certainly has implications for animal welfare, as such dogs are experiencing emotional distress/pain. In addition to the undesirability of this situation in itself, such discomfort experienced repeatedly may also lead to other behavioural or physical problems. It therefore is important for homeless persons to have access to resources that might mitigate situations where their pet is at risk of separation distress, and also to provide education to them regarding effective ways of addressing this issue.

An additional animal welfare issue that is specific to this population is thermal discomfort. Homeless pet owners who live in a vehicle or a tent must monitor their pet for possible heat stress or frost bite during inclement weather and prepare for access to resources such as indoor environments, fresh water, blankets, and dog booties/coats. Humane societies/shelters often have pamphlets available with information pertaining to pet care when weather is extreme.

Among the non-homeless population, there is a prevalent view that persists that homeless persons should not own pet dogs (Kuhlenbeck 2021). The findings of this study can raise public awareness regarding the welfare of these dogs. Findings from this study also suggest that often dogs belonging to a homeless person have a “life worth living” (Webster 2016).

## Supporting information

King et al. supplementary material King et al. supplementary material

## Supplementary material

The supplementary material for this article can be found at http://doi.org/10.1017/awf.2024.12.

## References

[r1] American Society for the Prevention of Cruelty to Animals (ASPCA) 2023 https://www.aspcapro.org/ (accessed 02/02/2024).

[r2] Bowlby J 1969 Attachment. Attachment and Loss, Volume 1: Loss. Basic Books: New York, NY, USA.

[r3] Cleary M, West S, Visentin D, Phipps M, Westman M and Vesk K 2021 The unbreakable bond: The mental health benefits and challenges of pet ownership for people experiencing homelessness. Issues in Mental Health Nursing 42: 741–746. 10.1080/01612840.2020.1843096 33196324

[r4] Clopper C and Pearson E 1934 The use of confidence or fiducial limits illustrated in the case of the binomial. Biometrika 26: 404–413. 10.2307/2331986

[r5] Coria-Avila G, Pfaus J, Orihuela A, Dominguez-Oliva A, Jose-Perez N, Astrid Hernandez L and Mota-Rojas D 2022 The neurobiology of behavior and its applicability to animal welfare: A review. Animals 12: 928. 10.33990/ani12070928 35405916 PMC8997080

[r6] Henwood B, Dzubur E, Rhoades H, St. Clair P and Cox R 2020 Pet ownership in the unsheltered homeless population in Los Angeles. Journal of Social Distress and Homelessness 30: 191–194. 10.1080/10530789.2020.1795791

[r7] Irvine L 2013a Animals as lifechangers: Pets in the redemption narratives of homeless people. Journal of Contemporary Ethnography 42: 3–30. 10.1177/0891241612456550

[r8] Irvine L 2013b My dog always eats first: Homeless people and their animals. Lynne Reinner Publishers Inc: Boulder, CO, USA.

[r9] Irvine L, Kahl K and Smith J 2012 Confrontations and donations: Encounters between homeless pet owners and the public. The Sociological Quarterly 53: 25–43. 10.1111/j.1533-8525.2011.01224.x 22329059

[r10] Kaldahl K 2023 Canine socialization: Tips to help fearful, shy, scared, and abused dogs. https://pethelpful.com/dogs/Socializing-Your-Dog-The-Basics (accessed 02/02/2024).

[r11] Kerman N, Lem M, Witte M, Kim C and Rhoades H 2020 A multilevel intervention framework for supporting people experiencing homelessness with pets. Animals 10: 1869. 10.3390/ani10101869 33066290 PMC7602009

[r12] King C, Smith T, Holman E, Serpell J and Grandin T 2021 Medical, behavioral, and abuse status characteristics: Predictors of perceived adoptability, appeal, and resource demands of shelter dogs. Anthrozoos 34. 10.1080/08927936.2021.1914435

[r13] Kuhlenbeck M 2021 Poor people are unjustly stigmatized for owning pets. *The Progressive Magazine.* https://progressive.org/latest/poor-people-stigmatized-owning-pets-kuhlenbeck-210723 (accessed 02/02/2024).

[r14] Laflamme D 1997 Development and validation of a body condition score system for dogs. Canine Practice 22: 10–15.

[r15] Mellor D 2016 Updating animal welfare thinking: Moving beyond the “Five Freedoms” toward “A life worth living.” Animals 6: 21. 10.3390/ani6030021 27102171 PMC4810049

[r16] Nagasawa M, Mogi K, and Kikasui T 2009 Attachment between humans and dogs. Japanese Psychological Research 51: 209–221. 10.1111/j.14685884.2009.00402.x

[r17] National Alliance to End Homelessness 2020 *National Alliance to End Homelessness. Keeping people and pets together.* https://endhomelessness.org/wp-content/uploads/2020/03/Keeping-People-and-Pets-Together-031220 (accessed 02/02/2024).

[r18] National Alliance to End Homelessness 2023 *State of Homelessness: 2023 Edition.* https://endhomelessness.org/homelessness-in-america/homelessness-statistics/state-of-homelessness/ (accessed 02/02/2024).

[r19] One Welfare 2023 https://www.onewelfareworld.org (accessed 02/02/2024).

[r20] Pinillos R 2018 One Welfare: A Framework to Improve Animal Welfare and Human Well-being. CABI Publishers: Wallingford, Oxon, UK.

[r21] Rhoades H, Winetrobe H and Rice E 2015 Pet ownership among homeless youth: Associations with mental health, service utilization, and housing status. Child Psychiatry Human Development 46: 237–244. 10.1007/s10578-014-0463-5 24728815 PMC4194276

[r22] Scanlon L, Hobson-West P, Cobb K, McBride A and Stavisky A 2021a Homeless people and their dogs: Exploring the nature and impact of the human-companion animal bond. Anthrozoos 34: 77–92. 10.1080/08927936.2021.1878683

[r23] Scanlon L, Hobson-West P, Cobb K, McBride A and Stavisky J 2021b Assessment of health and welfare in a small sample of dogs owned by people who are homeless. The Veterinary Record e776. 10.1002/vetr.776 34402075

[r24] Street Dog Coalition 2023 Street Dog Coalition: Fort Collins, Colorado, USA. https://www.thestreetdogcoalition.org (accessed 02/02/2024)

[r25] Webster D 2016 Animal welfare: Freedoms, dominions, and a “Life worth living.” Animals 6: 35. 10.3390/ani6060035 27231943 PMC4929415

[r26] Williams D and Hogg S 2016 The health and welfare of dogs belonging to homeless people. Pet Behavior Science 1: 23–30. 10.21071/pbs.v0i1.3998

[r27] Winkle M, Johnson A and Mills D 2020 Dog welfare, well-being, and behavior: Considerations for selection, evaluation, and suitability for animal-assisted therapy. Animals 10: 2188. 10.3390/ani10112188 33238376 PMC7700550

