# Supplementary material for: Physical and behavioural health of dogs belonging to homeless people
Source: Anim Welf. 2024 Feb 26;33:e11. doi: 10.1017/awf.2024.12 (PMC10951669; doi:10.1017/awf.2024.12)
Supplement: King et al. supplementary material [file S0962728624000125sup001.pdf]

Dog Survey #______

1. Dog sex: Male ☐ Female ☐

2. Spayed/Neutered: Yes ☐ No ☐ Dog's age: ________

3. Where did you obtain your dog? Dog shelter ☐ Friend/Family ☐ Found ☐ Other:_______

4. What is your current housing situation? __________________________________

5. Do you view your dog as a family member? Yes ☐ No ☐

***Note.* If respondent indicated that they own or rent a home in Q4 above, then END THE SURVEY.**

6. Why did you decide to take ownership of your pet dog and care for it?

   ____

7. Which do you most prefer? A dog for protection ☐ A dog for companionship/support ☐

8. Which do you most prefer? A more active dog ☐ A less active dog ☐

9. Have you ever rescued a dog who was previously abused? Yes ☐ No ☐

10. Did you or your family have a dog as a child? Yes ☐ No ☐

11. What behaviors would you like to see corrected in your dog?

    ____

12. Do you ever take your dog to a dog park? Yes ☐ No ☐

13. Who cares for your dog if you are temporarily unavailable to care for it?

    ____

14. Does your dog ever show distress if separated from you? Yes ☐ No ☐

15. If so, what distressful behaviours?

____

16. What type of food do you give to your dog?

____

17. What obstacles have you faced while owning your dog?

____

18. What emergencies, if any, have you had with your dog?

____

____

_____

19. How would you describe your dog's interaction with other dogs?

____

_____

20. What is the reaction of unfamiliar people toward your dog?

☐ mostly positive ☐mostly negative ☐mostly neutral

21. What one word best describes what your dog means to you?

____

<u>Visual assessment</u>

<u>OBSERVE the condition of the dog</u>. Place notes on the survey

A. Describe the dog's behaviour:

☐ Friendly, tail wagging

☐ Shy, fearful, or cowering

☐ Growling or teeth showing

☐ Neutral

☐ Other (describe):

____

B. What does the dog's physical body/health look like?

☐ Overweight

☐ Underweight

☐ Dirty

☐ Fleas, mites, hair loss?

Any visual evidence of dog being sick? (limp, swollen paw, red eye, etc.) Yes ☐ No ☐

C. **In your opinion, does the dog appear attached to the owner?** Yes ☐ No ☐

D. **Is the dog wearing a leash or collar?** Yes ☐ No ☐

Does the dog's collar have tags? Yes ☐ No ☐

E. **Breed** – Identify as best you can, since most will be mixed breeds (i.e. does the dog look like a pit bull, border collie, chihuahua, German shepherd, etc):

____

F. **Dog's size** Small (<20 lbs.)☐ Medium (20-40 lbs.)☐ Large (>40 lbs.)☐
